# Molecular study of hearing loss in Minas Gerais, Brazil^☆^

**DOI:** 10.1016/j.bjorl.2018.12.005

**Published:** 2019-02-20

**Authors:** Raíssa de Oliveira Aquino Schüffner, Karla Lima Nascimento, Fábio André Dias, Pedro Henrique Teodoro da Silva, Wrgelles Godinho Bordone Pires, Nilson Moreira Cipriano, Luciana Lara dos Santos

**Affiliations:** Universidade Federal de São João del-Rei, Divinópolis, MG, Brazil

**Keywords:** Hearing loss, GJB2, GJB6, Brazil, Perda auditiva, GJB2, GJB6, Brasil

## Abstract

**Introduction:**

Deafness is the most frequent sensory deficit in humans. Incidence is estimated at 4:1000 births in Brazil. Specific programs for clinical care of patients with hearing loss are still scarce in Brazil and the issue is an important public health problem.

**Objective:**

To determine the frequency of 35delG and D13S1830 mutations in *GJB2* and GJB6 *g*enes respectively in patients with non-syndromic sensorineural hearing loss from Minas Gerais, Brazil.

**Methods:**

This research involved 53 individuals, who were assessed by a questionnaire for predicting the possibility of non-syndromic deafness and for data collecting. Samples were tested for the presence of the 35delG mutation in *GJB2* gene and D13S1830 in *GJB6* gene by polymerase chain reaction and restriction enzyme digestion.

**Results:**

Epidemiological research has shown that the majority of the subjects are unaware of the etiology and the pathogenesis of hearing loss. In 9 patients (16.98%), 35delG mutation was found in heterozygosis and the allele frequency was estimate to be around 8.5%. Although 9.61% of the patients reported having some degree of consanguinity between the parents and 12.08% reported other cases of deafness in their families, this mutation was not found in homozygosis. The D13S1830 mutation was not found in this study.

**Conclusion:**

This research describes for the first time the frequency of the 35delG and D13S1830 mutation in hearing-impaired individuals from Minas Gerais, Brazil, and the collected data reinforce the need for further studies in this population due to heterogeneity of hearing loss.

## Resumo

**Introdução:**

A surdez é o déficit sensorial mais frequente em humanos. Estima-se que a incidência seja de 4:1.000 nascimentos no Brasil. Programas específicos para atendimento clínico de pacientes com perda auditiva são escassos no Brasil e a questão é um importante problema de saúde pública.

**Objetivo:**

Determinar a frequência das mutações 35delG no gene *GJB2* e D13S1830 no *GJB6* em pacientes deficientes auditivos de origem neurossensorial e não sindrômica de Minas Gerais, Brasil.

**Método:**

A pesquisa envolveu 53 indivíduos selecionados por meio de questionário o qual avaliou a possibilidade de surdez não sindrômica entre outros dados. As amostras foram testadas quanto à presença da mutação 35delG no gene *GJB2* e D13S1830 no gene *GJB6* por reação em cadeia da polimerase e digestão com enzima de restrição.

**Resultados:**

A pesquisa epidemiológica mostrou que a maioria dos indivíduos desconhece a etiologia da perda auditiva. Em 9 pacientes (16,98%), a mutação 35delG foi encontrada em heterozigose e a frequência alélica foi estimada em 8,5%. Embora 9,61% das pessoas tenham relatado algum grau de consanguinidade entre os pais e 12,08% relatassem outros casos de surdez em suas famílias, essa mutação não foi encontrada em homozigose. A mutação D13S1830 não foi encontrada neste estudo.

**Conclusão:**

Este trabalho descreve pela primeira vez a frequência da mutação 35delG e D13S1830 em deficientes auditivos de Minas Gerais, Brasil, e os dados coletados reforçam a necessidade de mais estudos nessa população devido à heterogeneidade da perda auditiva.

## Introduction

Deafness is the most frequent sensory deficit in humans, with an incidence that ranges from 1:300 to 1:1000 children. According to the World Health Organization (WHO), hearing loss affects about 10% of the world population.^1^ The frequency of deafness in Brazil is estimated at 4:1000 births. In Brazil environmental factors account for 80% of cases, and genetic causes are responsible by the other 20%.^2^, ^3^

Non-syndromic hereditary forms, which are not associated with other clinical manifestations, account for about 70% of cases and can be autossomal recessive (DFNB), autossomal dominant (DFNA), X-linked or mitochondrial. The recessive forms are more prevalent (75%–80% of cases) and tend to be more severe and often associated with defects in the cochlea, leading to the neurosensorial disability. The dominant forms (15%–20% of cases) are milder and contribute in most cases to post-lingual deafness progressive. The X-linked forms are rarer, as well as the mitochondrial form.^4^, ^5^, ^6^, ^7^, ^8^ The early identification of deafness with etiological diagnosis also enables early treatment, improving the prognosis. In Brazil, the specific programs for clinical care of patients with hearing loss are still scarce and this is an important public health problem. The research of non-syndromic Congenital Deafness with Guthrie test is not available at the Brazilian public health system (SUS, Unified Health System) and just Otoacoustic Emissions Test (OAE) are required, by law, in the newborns.^9^ Besides, molecular diagnosis is more restricted in cientific researches, and the high genetic heterogeneity of non-syndromic hearing loss is a complicating factor.^10^

More than 150 loci have been mapped and 93 genes have been identified for non-syndromic hearing loss,^11^ though the involved genes and frequencies of mutations are strongly influenced by the ethnic composition of the population.^12^ Mutations in three genes encoding connexin, *GJB2* (Cx 26), *GJB6* (Cx 30), and *GJB3* (Cx 31), were found to cause hearing impairment, and the occurrence in the first two are most frequently described.^2^ Variations in *GJB2* gene account for up to 50% of all genetically caused non-syndromic hearing loss cases in Caucasians.^13^ There are no reports on the frequency of the mutations in these genes in deafness population from Minas Gerais, Brazil and here we estimated the frequency of two frequently described mutations, 35delG in *GJB2* gene and D13S1830 in *GJB6* gene in unrelated hearing-impaired patients from Minas Gerais, Brazil.

## Methods

### Sample

We conducted a cross-sectional study, in which 53 disabled, 32 males and 21 females, all unrelated, were studied. We included in the study patients without medical confirmation about disability's cause. Therefore, were included patients with hearing impairment classified as unknown case, with no diagnostic hypothesis about the cause of hearing loss, absence of confirmation through specific tests and/or medical confirmation described in the report. There was no age restriction for participation in this study. All individuals are residents of the Midwest region of Minas Gerais. Patients were recruited from school Audio-Visual Assistance for Hearing Impaired (AAVIDA) and the Deaf Association, both from Divinópolis, Minas Gerais, Brazil.

This study was approved by the Ethics Committee on Human Research from Universidade Federal de São João Del Rei, Minas Gerais, with favourable opinion number 009/2011. All steps for clarification of the study were performed using the Brazilian Sign Language (Libras) and Portuguese language, so all participants have full autonomy in deciding their participation in research. Those individuals who have decided to participate signed an informed consent.

We used a questionnaire to collect data related to age of onset of hearing loss, presence of other cases in the family, consanguineous relationships in the family, manifestation of syndromes associated to hearing loss to confirming non-syndromic deafness and exclusion of environmental causes with medical confirmation: prenatal infections such as rubella or toxoplasmosis, postnatal infections, and exposure to ototoxic drugs.

### Molecular analysis

Blood samples (5 mL) were collected from the patients and genomic DNA isolated according to standard procedures.^14^ The amplification of the genomic region for analysis of the 35delG mutation in the *GJB2* gene was performed with primers previously described and the annealing temperature used was 65 °C.^2^ The PCR produces an 89 bp fragment which is digested by restriction enzyme BstNI. Individuals without the 35delG mutation have the restriction site for the enzyme BstNI and produce two fragments of 69 bp and 20 bp. The 35delG deletion abolishes the restriction site of the enzyme and consequently homozygous individuals for this mutation produce only a fragment of 89 bp and heterozygous individuals produce the three fragments described above.^2^

The D13S1830 mutation in the GJB6 gene was detected by standard PCR technique using three primers, F: (TTT AGG GCA TGA TTG GGG TGA TTT), R1: (CAC CAT GCG TAG CCT TAA CCA TTTT) and R2: (TCA TCG GGG GTG TCA ACA AACA).^2^ The annealing temperature was 62 °C. The primers R1 and R2 are located in the gene encoding connexin 30 protein, and primer F is located before the 342 kb region, which is deleted in mutant individuals. The reverse primer (R2), in turn, is located close to the breakpoint DNA in the presence of the mutation. Normal individuals who do not have the mutation will form 681 pb products amplified by primers R1 and R2. When the deletion occurs, the primer annealing region R2 is lost approaching the regions annealing of the primers F and R1 and amplify a fragment of 460 pb. Therefore, the heterozygous individuals have products with 681 pb and 460 pb and homozygous mutated only 460 pb fragment. Two mutations (35delG and D13S1830) were screened in all participants.

The PCR products were visualized on 8% polyacrylamide gel stained with silver nitrate. The DNA extraction product was stored in the freezer (mean −6 °C) and in a similar manner as the PCR products. All analyzes were carried out in a specialized laboratory in molecular biology of the Federal University of São João del-Rei, Divinópolis, MG, Brazil.

## Results

In this study, a total of 53 individuals with hearing impairment were evaluated. The age of patients ranged from 5 to 42 years old, with an average of 23.5 years.

Regarding the stage of hearing loss, 32.07% of the individuals presented manifestation at birth, 26.41% perceived the disability before 2 years old, and 11.32% after 2 years old. This information was not assessed for 30.18% of the individuals.

Among patients who answered questions about the etiology of deafness, nobody had medical report confirming the etiology of hearing loss. Patients with suspicions about the possible cause of disability but without medical confirmation corresponded to 60.37% of the sample; data were not available for 39.62%. The large number of missing data may be related to the lack of information of individuals on the probable cause of hearing disability.

Brazilian Sign Language (Libras) is a form of communication used by most participants, 86.79%. Only 9.43% use oral communication and data were not accessed for 3.77% of the sample.

In consanguinity analysis, 9.61% of people reported having some degree of consanguinity between the parents (confirmed only by simple genedogram) and 12.08% reported other cases of deafness in their families.

About molecular analysis, individuals with deafness of unknown etiology and who lived in the Midwest state of Minas Gerais, Brazil, were investigated for the presence of 35delG and D13S1830 mutations in *GJB2* and *GJB6* respectively. Of the 106 alleles present in the 53 individuals who underwent molecular analysis, nine were 35delG mutation. So, the allele frequency of 35delG mutation in the sample studied was 8.5%. All subjects with this mutation were heterozygous (16.98%). Even though patients reported the presence of consanguinity relationships in some families, homozygous individuals for this mutation were not found. The D13S1830 mutation was not found in any of the individuals tested.

## Discussion

Cases of pre-lingual deafness may be associated with environmental factors or genetic inheritance. Environmental factors in Brazil are among the major causes of hearing loss, and congenital rubella syndrome and neonatal anoxia are the most prevalent associated risk factors (33.5%), followed by those of unknown etiology (18.5%). In post lingual deafness cases the loss may be a consequence of the use ototoxic drugs, family history of hearing loss or in some cases can be considered idiopathic.^15^

The 35delG mutation in the *GJB2* gene is the main cause of genetic deafness in caucasian populations and it has been described in all studies conducted in other Brazilian regions.^13^, ^15^, ^16^ This mutation was found in heterozygosity in 16.98% of the sample studied. Recessive forms of hearing loss are the most prevalent in non-syndromic hereditary forms. When patients had the 35delG mutation in both alleles (homozygous), it is possible to affirm that hearing impairment manifested as a result of this genotype. However, when the mutation is in heterozygosis, as in most cases, it is necessary to do further analysis in the *GJB2* gene, to investigate a possible compound heterozygosis as a justification of the manifestation of deafness.

The frequency of 35delG mutation in a sample of hearing impairment from Minas Gerais state has been described for the first time in this study. This frequency is similar to others already described in populations from Southeastern Brazil (Table 1). Those that are not in agreement may be due to the sample size and the selection criteria of the sample.^2^, ^8^, ^17^, ^18^, ^19^ Despite the fact that D13S1830 is a mutation frequently described in Brazilian populations, in the sample from Minas Gerais this allele was not found. Some other studies also did not found this mutation in their analysis.^19^, ^20^, ^21^ Even though environmental causes of deafness may be highly frequent in Brazil, identification of the hidden genetic causes by the molecular tests is important. The genetic test is useful for early diagnosis to achieve the objectives in the rehabilitation for deaf people and genetic counseling. Epidemiological research, as described here, has shown that the majority of the subjects are unaware of the etiology of deafness. Some of them suspect of the cause, but no individual has a medical report, confirming the lack of data regarding the etiology and the pathogenesis of hearing loss. All these aspects involved in the etiologic understanding of deafness is important for providing a better prognosis; stimulation of speech and weakness of the lower dyad hearing-speech, contributing to a better quality of life for individuals with hearing loss genetic non-syndromic and their families.^17^

**Table 1:** Frequency of 35delG mutation in previous studies performed in Brazilian populations.

Region	Sample population	Allele 35delG	References
Minas Gerais State	53 patients with nonsyndromic hearing loss	8.5%	Present study
São Paulo State	300 patients with nonsyndromic hearing loss	9.8%	^17^
São Paulo State	180 patients with nonsyndromic hearing loss	13.30%	^18^
São Paulo State	33 patients with congenital nonsyndromic sensorineural hearing loss	21.21%	^8^
Espirito Santo State	77 patients with nonsyndromic hearing loss	7.8%	^2^
North Brazil	77 patients with nonsyndromic hearing loss	5.19%	^19^

Although variations in *GJB2* gene almost exclusively cause prelingual autosomal recessive hearing loss, this disorder is very heterogeneous.^13^ Other mutations, or other genes not studied here, might be involved in the disability pathogenesis. The sequencing of the *GJB2* gene could be useful to identify the second mutant allele and in spite of sequencing of the *GJB6* gene was not reported in Brazilian populations until now, it would be interest to identify other possible frequent mutation associated to the hearing disability.

## Conclusion

This study describes for the first time the molecular investigation of two genes related to the hearing loss in Minas Gerais population. The allele frequency of 35delG mutation in the sample studied was 8.5% and the D13S1830 mutation in GJB6 gene was not found in the patients investigated. These results reinforce the need for further studies about the investigation of deafness in Brazil, in order to improve public health issues and genetic counseling. Genetic and epidemiological information make possible etiologic identification, genetic counseling for families and better assessment to the possibility of treatment.

## Conflicts of interest

The authors declare no conflicts of interest.
